# SEOM Clinical Guideline for the diagnosis and treatment of esophageal cancer (2016)

**DOI:** 10.1007/s12094-016-1577-y

**Published:** 2016-11-29

**Authors:** M. Martin-Richard, R. Díaz Beveridge, V. Arrazubi, M. Alsina, M. Galan Guzmán, A. B. Custodio, C. Gómez, F. L. Muñoz, R. Pazo, F. Rivera

**Affiliations:** 1 Servicio de Oncología Médica, Medical Oncology Department, Hospital de la Santa Creu I Sant Pau, Sant Antoni Maria Claret, 167, 08025 Barcelona, Spain; 2 Medical Oncology Department, Hospital Universitari I Politècnic la Fe, Valencia, Spain; 3 Medical Oncology Department, Hospital de Basurto, Bilbao, Spain; 4 Medical Oncology Department, Hospital Vall d´Hebron, Barcelona, Spain; 5 Medical Oncology Department, Hospital Durán i Reynals (ICO), L’Hospitalet de Llobregat, Spain; 6 Hospital Universitario la Paz, Madrid, Spain; 7 Medical Oncology Department, Hospital Universitario 12 de Octubre, Madrid, Spain; 8 Medical Oncology Department, Hospital Universitario Ramón y Cajal, Madrid, Spain; 9 Medical Oncology Department, Hospital Universitario Miguel Servet, Zaragoza, Spain; 10 Medical Oncology Department, Hospital Universitario Marqués de Valdecilla, Santander, Spain

**Keywords:** Esophageal cancer, Diagnosis, Treatment

## Abstract

Esophageal cancer (EC) is an aggressive tumor that represents the 6th most common cause of cancer death worldwide. The estimated incidence in Spain is 2090 cases/year. Two main pathological subtypes exist, squamous cell carcinoma and adenocarcinoma. The main differences between them are localization and underlying factors which are the principal cause of the recent incidence changes observed in west countries. Staging techniques and treatment options which combine surgery, chemotherapy and radiotherapy, reflected the high complexity of the EC management. An undeniably multidisciplinary approach is, therefore, required. In this guide, we review the status of current diagnosis and treatment, define evidence and propose recommendations.

## Introduction, epidemiology, localization, histology and molecular biology

Esophageal cancer (EC) is the 6th leading cause of death from cancer and the 8th most common cancer in the world. The 5-year survival rate is around 15–25%; best results related to early stages. In Spain in 2012, there was an estimated incidence of 2090 new cases with 1728 deaths [1].

There are two main types of EC: the squamous cell carcinoma (SCC), typically found in the upper-middle esophagus, and the adenocarcinoma (ADC), usually in the lower esophagus. While SCC dominates worldwide, the ADC is more frequent in the developed countries, and its incidence has been increasing steadily in the past four decades. The EC is about 2–4 times more common in men than in women. Different risk factors have been described both for SCC and for ADC. While Tobacco, alcohol, mate, nitrogenous compounds, chewing betel nut and deficits of minerals and vitamins have been associated with SCC, tobacco, gastro-esophageal reflux, Barrett’s esophagus, obesity and low-fiber diet have been linked with ADC [2]. Non-steroidal anti-inflammatory drugs and proton-pump inhibitors have been proposed as protective factors in ADC.

With reference to the molecular biology of EC, the recent analysis of the TCGA (The Cancer Genome Atlas) has described the expression of 2962 genes (2081 up regulated and 881 down regulated) and 45 microRNAs (25 up regulated and 20 down regulated) intrinsic of EC; most of the mispregulated genes were involved in cellular signaling pathways and in tumorigenesis [3].

## Diagnosis and staging

The diagnosis should be made from an endoscopic biopsy and the histology to be reported according to World Health Organization (WHO) Criteria.

Once the pathologic diagnosis is established, accurate clinical staging is critical for estimating prognosis and selecting the appropriate treatment strategy. The following staging work-up is recommended:

- WHO performance status (PS), physical examination and comprehensive geriatric assessment in the elderly.
- Nutritional assessment and counseling (Evidence: moderate-quality; Recommendation: strong).
- Blood counts, liver and renal function tests.
- Computed tomography (CT) scan of chest and abdomen (E: moderate; R: strong).

In candidates for surgical resection or radical treatment, the following tests should be considered:

- Endoscopic ultrasound (EUS) is the most accurate technique for loco regional staging with an overall accuracy for tumor (T) and node (N) staging of 80–90%. The addition of fine needle aspiration (FNA) to EUS increases the accuracy of the lymph node involvement diagnosis (E: moderate; R: strong).
- 18F-FDG positron emission tomography (PET) or PET-CT (preferred) may detect radio graphically occult distant metastases in 10–20% of patients [4]. (E: moderate; R: strong).
- Bronchoscopy in case of tumors at or above the tracheal bifurcation. (E: moderate; R: strong).
- In locally advanced (T3/T4) distal esophageal or esophagogastric junction (EGJ) adenocarcinomas, staging laparoscopy and peritoneal cytology may rule out occult peritoneal metastases, which are found in about 15% of patients [5] (E: moderate; R: weak).

Staging is performed according to the 2010 UICC-AJCC system (7th edition) (Table 1) and grouped into separate stage categories in accordance with histology (Table 2) [6].

**Table 1:** TNM staging for esophageal and esophagogastric junction (EGJ) cancer (AJCC/UICC 7th edition)

Primary tumour (T)^a^	
Tx	Primary tumour cannot be assessed
T0	No evidence of primary tumour
Tis	High-grade dysplasia
T1	Tumour invades lamina propia, muscularis mucosae or submucosa
T1a	Tumour invades lamina propia or muscularis mucosae
T1b	Tumour invades submucosa
T2	Tumour invades muscularis propia
T3	Tumour invades adventitia
T4	Tumour invades adjacent structures
T4a	Resectable tumour invading pleura, pericardium or diaphragm
T4b	Unresectable tumour invading other adjacent structures, such as aorta, vertebral body, trachea, etc.
Regional lymph nodes (N)^b^	
Nx	Regional lymph nodes cannot be assessed
N0	No regional lymph node metastasis
N1	Regional lymph node metastasis involving 1–2 nodes
N2	Regional lymph node metastasis involving 3–6 nodes
N3	Regional lymph node metastasis involving 7 or more nodes
Distant metastasis (M)	
M0	No distant metastasis (no pathologic M0; use clinical M to complete stage group)
M1	Distant metastasis
Histologic grade (G)	
GX	Grade cannot be assessed-stage grouping as G1
G1	Well differentiated
G2	Moderately differentiated
G3	Poorly differentiated
G4	Undifferentiated-stage grouping as G3 squamous

^a^At least maximal dimension of the tumour must be recorded and multiple tumours require the T(m) suffix. High-grade dysplasia (HGD) includes all non-invasive neoplastic epithelia that was formerly called carcinoma in situ, a diagnosis that is no longer used for columnar mucosae anywhere in the gastrointestinal tract

^b^Number must be recorded for total number of regional nodes sampled and total number of reported nodes with metastasis

**Table 2:** Stage grouping according to histology

Squamous cell carcinoma^a^						Adenocarcinoma				
Group	*T*	*N*	*M*	Grade	Tumour location^b^	Group	*T*	*N*	*M*	Grade
0	Tis (HGD)	N0	M0	1	Any	0	Tis (HGD)	N0	M0	1, X
IA	T1	N0	M0	1, X	Any	IA	T1	N0	M0	1-2, X
IB	T1 T2-3	N0 N0	M0 M0	2-3 1, X	Any Lower, X	IB	T1 T2	N0 N0	M0 M0	3 1-2, X
IIA	T2-3 T2-3	N0 N0	M0 M0	1, X 2-3	Upper, middle Lower, X	IIA	T2	N0	M0	3
IIB	T2-3 T1-2	N0 N1	M0 M0	2-3 Any	Upper, middle Any	IIB	T3 T1-2	N0 N1	M0 M0	Any Any
IIIA	T1-2 T3 T4a	N2 N1 N0	M0 M0 M0	Any Any Any	Any Any Any	IIIA	T1-2 T3 T4a	N2 N1 N0	M0 M0 M0	Any Any Any
IIIB	T3	N2	M0	Any	Any	IIIB	T3	N2	M0	Any
IIIC	T4a T4b Any	N1-2 Any N3	M0 M0 M0	Any Any Any	Any Any Any	IIIC	T4a T4b Any	N1-2 Any N3	M0 M0 M0	Any Any Any
IV	Any	Any	M1	Any	Any	IV	Any	Any	M1	Any

*HGD* high-grade dysplasia

^a^Or mixed histology including a squamous component or NOS

^b^Location of the primary cancer site is defined by the position of the upper (proximal) edge of the tumour in the oesophagus

## Treatment

Initial treatment approaches for EC depend on several factors, and these patients should all be discussed in a multidisciplinary setting (E: moderate; R: strong) (Table 3).

**Table 3:** Diagnosis and treatment evidences and recommendations

General	Details	Evidence	Recommendation
**Diagnosis and staging**			
PS evaluation		Moderate	Strong
Physical examination		Moderate	Strong
Geriatric assessment in elderly		Moderate	Strong
Nutritional assessment		Moderate	Strong
Blood counts, liver and renal functional tests		Moderate	Strong
Computed chest and abdomen tomography (CT scan)		Moderate	Strong
Endoscopic ultrasound (EUS) +/− fine needle aspiration (FNA)		Moderate	Strong
18F-FDG positron emission tomography (PET) or PET-CT (preferred)		Moderate	Strong
Bronchoscopy	Tumors at or above the tracheal bifurcation	Moderate	Strong
Staging laparoscopy and peritoneal cytology	In locally advanced (T3/T4) distal esophageal ADC	Moderate	Weak
**Treatment**			
Early stage (Tis and T1-2)			
Tis	Ablation	Low	Strong
	Surgery	Low	Weak
T1a N0 (<2 cm, well or mod)	Endoscopic resection	Low	Strong
	Surgery	Low	Weak
T1b-2N0	Surgery	Moderate	Strong
Locally advanced disease (T3-4N0 and T1b-T4aN+)			
Cervical esophagus			
	Definitive CRT (cisplatin-FU + RT)	High	Strong
Thoracic esophagus			
SCC	Preoperative CRT (cisplatin-FU or TXL-carboplatin or carbolatin–FU + RT)	Moderate	Strong
	Definitive CRT	Moderate	Weak
	Preoperative CT	Low	Weak
	NO Postoperative CT	High	Strong
ADC	Preoperative CRT	Moderate	Strong
	Perioperative CT (distal tumor)	Moderate	Strong
Locally advanced disease, unresectable (T4bNx)			
Fit patients	Definitive CRT Cisplatin-FU + RT	High	Strong
Unfit patients	Other CT (oxaliplatin-FU, or carboplatin–placitaxel)	Moderate	Strong
Metastatic disease			
PS 0-2	1st line (platinum-Fluo)	High	Strong
	2nd line	Low	Weak
PS > 2	Supportive care	Moderate	Strong

*SCC* squamous cell carcinoma, *ADC* adenocarcinoma, *CRT* chemoradiotherapy (*CT* chemotherapy and *RT* radiotherapy), *TXL* pa clitaxlel *FU* fluorouracil, *Fluo* fluoropyrimidine

Adequate evaluation of comorbidities and management and treatment of cancer complications play an important role in these patients. Nutritional support is required for patients with significant dysphagia and weight loss. Oral supplementation, nasogastric tube or percutaneous endoscopic gastrostomy may be considered for preoperative nutritional support as well as for cervical tumors or non-surgical candidates receiving definitive chemo radiation. (E: moderate; R: strong).

Superficial cancers and high-grade dysplasia of the esophagus may be treated by endoluminal therapy. This may be by ablation of the mucosa using a variety of techniques (no specimen for pathological examination) or by endoscopic mucosal resection (EMR) or endoscopic sub mucosal dissection (ESD) (can be used to both stage and treat early cancer).

Surgery is an accepted single-modality therapy for patients with early localized disease or for patients who may not tolerate combined-modality therapy. Surgical options include transhiatal esophagectomy and transthoracic approaches, with selection based on surgical expertise, the goal of reducing risk of complications, individual anatomy and patient preference. No approach has been demonstrated to lead to superior cure rates [7]. In addition to operator technique, intensive care unit management and early detection of complications likely play a role in these differential outcomes. Esophagectomy should be performed in high-volume esophageal cancer centers by experience surgeons [8] (E: moderate; R: strong). The optimum number of nodes removed in the lymphadenectomy is not established although in retrospective studies a greater extension of lymphadenectomy is related to better outcome. Minimally invasive esophagectomy is another surgical option which has shown similar efficacy to open approaches but with less surgical complications [9] (E: moderate; R: strong).

Combined chemoradiation leads to prolonged median survival and long-term survival compared with radiation alone when used as a definitive non operative approach [10]. The standard treatment is external beam radiotherapy for a total dose of 50.4 Gy (strong recommendation; high-quality evidence). There is no demonstrated benefit to escalation of radiation dose in this setting to 64.8 or to the use of twice-daily irradiation [11] **(**E: high R: strong).

### Early disease (Tis-T2N0M0)

#### Endoscopic treatments

Endoscopic resection (mucosal or sub mucosal resection) with or without endoscopic ablation (cryoablation or radiofrequency) may be used in T1a tumors (less than or equal to 2 cm, and well or moderately differentiated carcinoma) with less morbidity than surgery. Although no randomized studies have compared these two strategies, retrospective series show that endoscopic procedures are effective treatment options. Ablation alone may be an appropriate treatment for patients with Tis tumors (E: low; R: strong).

#### Surgery

Esophagectomy is indicated for patients with T1a tumors with extensive carcinoma in situ, lesions larger than 2 cm, high-grade carcinomas and positive deep margins after endoscopic resection or linfovascular invasion. Moreover, surgery remains the first treatment of choice in all T1b-T2N0M0 tumors [12] (E: moderate; R: strong).

### Locally advanced disease (T3-4N0 and T1-4aN+, *M0*)

#### Cervical esophagus

Definitive chemoradiation with Cisplatin and 5-FU is the standard of treatment in this clinical setting (E: high; R: strong).

#### Thoracic esophagus

Multimodal approach is indicated in operable patients with locally advanced esophageal cancer.

### Squamous cell carcinoma

Preoperative chemoradiation followed by surgery is the most common approach for patients with resectable esophageal cancer. Different meta-analyses have suggested that preoperative chemoradiation based on Cisplatin and 5FU plus surgery significantly improved survival, compared with surgery alone [13]. The phase III CROSS study showed that preoperative chemoradiation (carboplatin plus paclitaxel) improved OS and DFS compared to surgery in patients with T2-3, N0-1, M0 neoplasias (median survival 49 vs. 24 months) [14]. However, this approach is not a standard therapy in stage I–II because the FFCD 9901 study did not improve OS with chemoradiation therapy with Cisplatin and fluorouracil compared with surgery (3-year OS 47.5 and 53%, *p* = 0.94). In this study the postoperative mortality rate was 11% for chemoradiation compared to 3.4% for surgery alone (*p* = 0.049) [15]. (E: moderate; R: strong).

Definitive Chemoradiotherapy: Two randomized trials [16, 17] did not confirm a survival benefit with surgery added to potentially curative chemoradiation (defined as a higher radiation dose of 60–66 Gy) in SCC, although there is a significant local control benefit. Although, both studies have important limitations (suboptimal design, high treatment-related mortality and poor accrual) definitive chemoradiotherapy can be considered an option in SCC (E: moderate; R: insufficient). The benefit/risk balance between surgery and close surveillance should be discussed in a committee, considering each case individually (E: moderate R: strong) [18].

Preoperative Chemotherapy: Preoperative chemotherapy adds a small but significant benefit over surgery alone for all types of esophageal cancer, though it is stronger for ADC [13] (E: moderate; R: weak). A small randomized phase II study [19] compared neoadjuvant chemotherapy with chemoradiotherapy. The corresponding median OS or DFS was not different; however, the pathological response rate and R1 response rate favored chemoradiation arm (E: low).

Postoperative chemoradiation: the efficacy of this approach has not been demonstrated in randomized trial in patients with EC (E: high; R: strong).

### Adenocarcinoma

Preoperative chemoradiation (as described above) and perioperative chemotherapy are both accepted strategies in adenocarcinoma of esophageal cancer. Preoperative chemotherapy can be considered with the remarks described above (E: moderate; R: strong).

Perioperative Chemotherapy: perioperative chemotherapy approach may be offered to patients with resectable ADC of the lower esophagus. Two phase III studies showed OS and PFS benefit over surgery [20, 21]; these studies were mainly designed for gastric or EGJ cancer, but also included a small proportion of patients with adenocarcinoma of the lower esophagus (E: moderate; R: strong).

Even after complete tumor response to preoperative therapy, patients with ADC should proceed to surgery [21] (E: high; R: strong).

## Treatment for locally advanced unresectable oesophageal cancer (T4b)

The 7th AJCC/UICC Edition [6] subclassifies T4 esophageal tumors in T4a and T4b. T4b tumors are those that invade adjacent structures such as aorta, vertebral body or trachea and are considered unresectable.

These patients are underrepresented in most clinical trials and there are few clinical trials specially focused on this subgroup. However, based on the available data we can consider the following:

1. Definitive Chemoradiotherapy(CRT) in locally advanced disease is better than radiotherapy(RT) alone [10] (E: high; R:strong).
2. In nonsurgical approach, higher radiation doses higher than 50.4 Gy did not increase survival or local/regional control in a randomized comparison [11].
3. In SCC, in cases of response to neoadjuvant Chemoradiotherapy, further continuation of chemoradiation (increasing radiotherapy up to 60 Gy) resulted in equivalent overall survival compared with surgery, albeit that the non-operative strategy was associated with higher local tumor recurrence [16, 17].
4. Several new strategies such as upfront chemotherapy or changes in the chemotherapy regimen (i.e., Taxane-based scheme, FOLFOX or addition of Cetuximab) have been tested in prospective randomized trials showing no improvement in overall outcomes [22–25].

Given the above, definitive Chemoradiotherapy along with four courses of Cisplatin and 5-fluorouracil plus 50–50.4 Gy still remains the gold standard in unresectable (T4b) tumors. Increased radiation doses up to 60 Gy may be an option in some cases [16, 17] (E: high; R: strong).

## Treatment for non-metastatic disease in unfit patients

For a patient unable to undergo surgery, but able to tolerate chemotherapy plus radiotherapy, different regimens based on Oxaliplatin/Fluoropyrimidine [24] or carboplatin/paclitaxel [26] combinations may be an alternative to “classical” Cisplatin/5-fluorouracil schedule, due to their favorable toxicity profile (E:moderate; R: strong). If a patient is unable to tolerate combined therapy, chemotherapy alone is an option. Palliative radiotherapy or best supportive care is the appropriate option for non-surgical candidates who are unable to tolerate chemotherapy or chemoradiation.

## Assessment of response and follow up after definitive chemoradiotherapy

Assessment of response after treatment can include CT scan and esophagogastroscopy plus biopsies. PET scan can be useful in the evaluation of residual disease. The role of early metabolic response (PET scan) is investigational.

Follow up after treatment is controversial since very limited data are available. According to the NCCN guidelines (v 3.2015) it can include history and physical examination every 3–6 months for 1–2 years, every six months for 3–5 years and then annually. CBC, serum chemistry endoscopy with biopsy and imaging studies should be obtained as clinically indicated. Continuous nutritional counselling is advisable. In addition, some patients may require dilatation of an anastomotic or a chemoradiation-induced stricture.

### Metastatic esophageal carcinoma: chemotherapy

Despite differences in the biology [27], metastatic SCC and ADC EC are treated similarly. A first-line treatment with Cisplatin or Oxaliplatin combined with fluorouracil or Capecitabine can improve survival (E: high; R: strong) [28]. Best supportive care should be offered to all metastatic esophageal cancer patients since the first visit. In very good performance status patients addition of Epirrubicin and Taxanes could offer some additional benefit in GEJ adenocarcinomas [29, 30] (E: moderate; R: weak). Nonetheless, a less toxic two-drug regimen is usually preferred for patients with metastatic disease.

When progression occurs, the role of second-line chemotherapy in esophageal cancer is controversial since no randomized phase III trials have been done in this clinical setting, in either SCC or ADC, and there is only scarce data for its clinical effectiveness [31] (E: low; R: weak). The evidence of positive results, with improvement in overall survival, from second-line therapy in gastric cancer cannot be extrapolated to esophageal cancer in view of the clinical and biological differences between both tumor locations.

### Metastatic esophageal carcinoma: new targeted drugs

Recent insights into the molecular mechanism of esophageal cancer have led to the development of various targeted agents in this disease. Specifically, EGFR, HER2, VEGR and c-MET were shown in preclinical models as valuable targets for esophageal cancer. Despite signs of efficacy in early phase clinical trials, results with different anti-EGFR, anti-VEGFR and Anti-cMET/HGF agents have been unsuccessful so far [32–35] and these agents cannot be recommended at this time. Only Trastuzumab, antiHER2 directed monoclonal antibody, has been shown to improve overall survival in the 10–15% of patients with HER2 positive advanced gastric or gastro esophageal adenocarcinomas [36] (E: high; R: strong). Unfortunately, these positive results were not replicated in recent trials with other antiHER2 agents (Lapatinib, T-DM1). Novel strategies with agents targeting FGFR or Hedgehog pathways, poly (adenosine diphosphate-ribose) polymerase inhibitors or next-generation immune checkpoint antibodies are undergoing investigation in early phase trials that include esophageal cancer patients, with preliminary data demonstrating for immune checkpoint inhibitors manageable toxicity and promising antitumor activity in heavily treated patient.

Evidence to date shows that molecularly unselected patient cohorts derive no benefit from targeted therapies [37]. This way future research should focus on preselected molecular subgroups of patients with this disease.

### Supportive care and palliation

Patients with esophageal cancer refractory to the standard anticancer treatment or those who are medically unfit for any therapy (performance status >2) require focusing our efforts on the relief of symptoms and the improvement in quality of life [38]. An adequate caloric intake should be maintained in these patients primarily in the multimodality treatment scenario. Oral supplementation (preferred), feeding tubes for enteral nutrition or total parenteral nutrition are the main options (E: moderate; R: strong) Malignant dysphagia is one of the major issues in esophageal cancer. The insertion of endoluminal stents, the administration of palliative external beam radiation therapy and brachytherapy are the preferred options to alleviate dysphagia (E: high; R: strong); finally, pain, nausea and vomiting should be treated according to specific guidelines. (E: high; R: strong).
